# Constrained Nuclear-Electronic Orbital Theory for Quantum Computation

**DOI:** 10.1021/acs.jctc.5c00815

**Published:** 2025-08-12

**Authors:** Tanner Culpitt, Zehua Chen, Fabijan Pavošević, Yang Yang

**Affiliations:** † Theoretical Chemistry Institute and Department of Chemistry, 5228 University of Wisconsin-Madison, 1101 University Avenue, Madison, Wisconsin 53706, United States; ‡ Algorithmiq Ltd., Kanavakatu 3C, FI-00160 Helsinki, Finland

## Abstract

Quantum computing offers promising advantages for computational chemistry, particularly through algorithms that efficiently model correlated methods. In this work, we apply quantum computing techniques to the constrained nuclear-electronic orbital (CNEO) framework, which enables the inclusion of nuclear quantum effects in chemical simulations while preserving a well-defined molecular structure. We present the development and implementation of two correlated wave function methods within this framework: CNEO full configuration interaction (CNEO-FCI) and CNEO unitary coupled-cluster with singles and doubles (CNEO-UCCSD), with the latter solved using the variational quantum eigensolver algorithm. These methods were applied to the hydrogen isotopologues H_2_, HD, and D_2_ and used to calculate potential energy surfaces, equilibrium geometries, harmonic vibrational frequencies, and von Neumann entropies. The CNEO-UCCSD results are in excellent agreement with CNEO-FCI, recovering over 99% of the correlation energy and accurately capturing geometries and vibrational frequencies. Additionally, we observe a strong connection between CNEO-FCI and CNEO-UCCSD energy and entropy differences as a function of bond length, highlighting the role of quantum entanglement in molecular dissociation. These results demonstrate the viability of CNEO-based quantum algorithms for capturing nuclear quantum effects and lay the groundwork for future quantum simulations and entropy analysis of multicomponent systems within the CNEO paradigm.

## Graphical abstract

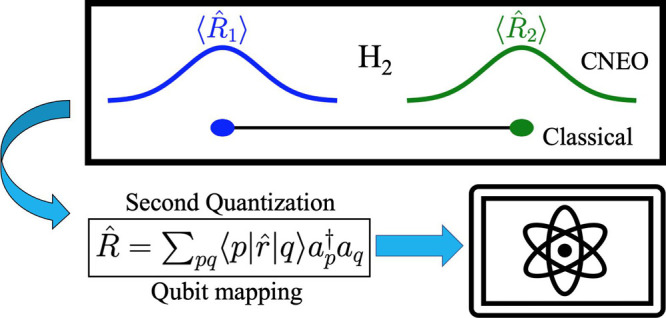

## 1 Introduction

Quantum computing shows promise in many areas of science, including chemistry,^1–4^ biology,^5–7^ and physics.^8–10^ So much attention has been given to quantum algorithms for solving computational problems because of the efficiency that can potentially be leveraged by quantum computers. For example, in the context of theoretical chemistry, exact solutions to the Schrödinger equation such as full configuration interaction (FCI) scale very poorly on classical machines. The FCI Hilbert space grows exponentially with system size, and solutions to the FCI problem are therefore only feasible for small molecules with small basis sets. Meanwhile, quantum algorithms such as the phase estimation algorithm (PEA)^11–13^ can scale polynomially with system size.

A significant portion of the development of quantum algorithms for computational chemistry has been focused on the electronic problem. However, there has recently been interest in multicomponent methods in quantum computing.^14–18^ Here, “multicomponent” refers to systems where more than one type of particle is treated quantum mechanically,^19–28^ with one commonly used framework being the nuclear-electronic orbital (NEO) framework. In NEO, orbitals are used to describe both electrons and nuclei (most commonly light nuclei), and the coupled nuclear-electron equations are solved simultaneously.

However, one consequence of simultaneously treating the electrons and nuclei quantum mechanically is the conceptual erosion of molecular structure and the attendant framework of the potential energy surface (PES).^29^ Calculations yield states for the joint electron–nuclear quantum system that depends only on the classical nuclear positions, thereby losing the classical degrees of freedom associated with the quantum nuclei. Within this framework, because of the small mass difference between classical and quantum nuclei, the dynamics are often highly nonadiabatic.^30,31^ In order to address these challenges, the constrained nuclear-electronic orbital (CNEO) framework was recently developed by the Yang group.^32,33^

The CNEO method is both theoretically and practically distinct from other orbital-based multicomponent methods in that CNEO retains the conventional picture of molecular structure by using the expectation value of quantum nuclei as the parametric variable of interest upon which the PES depends.^32,33^ Additionally, CNEO makes the distinguishable particle approximation between quantum nuclei, which is physically motivated by the fact that quantum nuclei are localized in nearly all chemical systems of interest. Notably, distinguishability also eliminates the need for fermionic or bosonic statistics when considering the nuclei, and has previously been used in the multicomponent quantum phase estimation algorithm.^14^ The CNEO method allows for nuclear quantum effects, particularly nuclear quantum delocalization effects, to be directly incorporated into the calculation of chemical properties, such as geometries,^33–35^ spectra,^36–39^ and reaction rates,^40,41^ while being able to use computational machinery that requires a surface, such as harmonic analysis,^42^ ab initio molecular dynamics (AIMD),^43,44^ and nonadiabatic dynamics.^45^

With these capabilities in mind, we herein develop quantum algorithms for the implementation of the CNEO method on quantum computers. Specifically, we focus on the development of CNEO unitary coupled-cluster (CNEO-UCC) solved via the variational quantum eigensolver (VQE) algorithm.^46–48^ The VQE algorithm is a hybrid quantum-classical algorithm that uses a classical machine for parameter optimization and a quantum machine for state preparation and energy measurement. Because there is a well-defined PES in the CNEO paradigm, a hybrid approach to molecular dynamics calculations with quantum computers can also be used, where quantum computation is used for the generation of the PES and classical computation is used for the propagation of the equations of motion. We note that such a scheme has been previously utilized in the conventional electronic structure case for AIMD.^49^ The advantage and novelty in our approach over standard AIMD is that CNEO inherently includes nuclear quantum effects, enabling more accurate predictions for systems where classical nuclear treatments fail. For these reasons, CNEO is a desirable avenue to pursue for multicomponent quantum computing.

This work is organized as follows. Section 2 contains theoretical background pertaining to CNEO for both CNEO Full Configuration Interaction (CNEO-FCI) and CNEO Unitary Coupled-Cluster (CNEO-UCC). Section 3 presents PESs, harmonic frequencies, and von Neumann entropies for the H_2_, HD, and D_2_ molecules. Summary and future directions are given in Section 4.

## 2 Theory

We consider molecular systems comprised of electrons, distinguishable quantum nuclei, and *N*_nuc_ classical nuclei ***R***_*J*_. Superscripts and subscripts γ, η, κ refer to a generic particle type. The set of all orbital indices for particle type γ is denoted *S*_γ_, while the occupied and virtual subsets of *S*_γ_ are given by *S*_γ_^o^ and *S*_γ_^v^, respectively. The set containing classical nuclear indices is denoted *S*_R_. When referring specifically to electrons, the superscript or subscript “e” will be used.

### 2.1 Second-Quantized Hamiltonian

As mentioned previously, CNEO assumes distinguishability of quantum nuclei, which is physically motivated by their localized character. Here, “distinguishable” means that every *individual* quantum particle that is not an electron is distinguished from all other particles. For example, suppose we have a system comprised of *N*_e_ electrons and *N*_p_ quantum protons. Then this system contains *N*_p_ + 1 particle types rather than just two, because we have assumed all quantum nuclei are distinguishable. Another consequence of distinguishability is that there are exchange interactions only between the electrons and none between the nuclei. The form of the Hamiltonian in second quantization is then (in atomic units)

$$\hat{H}=\sum_{\gamma }\sum_{pq\in S_{\gamma }}h_{pq}^{\gamma }a_{p}^{†}a_{q}+\frac{1}{2}\sum_{pqrs\in S_{e}}g_{pqrs}^{\mathrm{ee}}a_{p}^{†}a_{r}^{†}a_{s}a_{q}+\sum_{\eta >\gamma }\sum_{pq\in S_{\gamma }}\sum_{rs\in S_{\eta }}v_{pqrs}^{\gamma \eta }a_{p}^{†}a_{q}a_{r}^{†}a_{s}+V_{\mathrm{nuc}}$$ 1

The one-particle and two-particle integrals are given by

$$h_{pq}^{\gamma }=\int dx^{\gamma }\phi_{p}^{*}\left(x^{\gamma }\right)\left(-\frac{1}{2m_{\gamma }}\nabla^{2}+\underset{J\in S_{R}}{\sum }\frac{Z_{J}Q_{\gamma }}{\left|r^{\gamma }-R_{J}\right|}\right)\phi_{q}\left(x^{\gamma }\right)$$ 2

$$g_{pqrs}^{\mathrm{ee}}=\int dx_{1}^{e}dx_{2}^{e}\frac{\phi_{p}^{*}(x_{1}^{e})\phi_{q}(x_{1}^{e})\phi_{r}^{*}(x_{2}^{e})\phi_{s}(x_{2}^{e})}{\left|r_{1}^{e}-r_{2}^{e}\right|}$$ 3

$$v_{pqrs}^{\gamma \eta }=Q_{\gamma }Q_{\eta }\int dx^{\gamma }dx^{\eta }\frac{\phi_{p}^{*}\left(x^{\gamma }\right)\phi_{q}\left(x^{\gamma }\right)\phi_{r}^{*}\left(x^{\eta }\right)\phi_{s}\left(x^{\eta }\right)}{\left|r^{\gamma }-r^{\eta }\right|}$$ 4

In eqs 2 – [Disp-formula eq4], ***x*** ^γ^ is a mixed space-spin coordinate for particle γ, ***r*** ^γ^ is the corresponding spatial component, *Q*_γ_ is the charge of particle γ, *m*_γ_ is the mass of particle γ, and *Z*_*J*_ is the charge of classical nucleus *J*. The repulsion between the classical nuclei is given by

$$V_{\mathrm{nuc}}=\sum_{K>J\in S_{R}}\frac{Z_{J}Z_{K}}{\left|R_{J}-R_{K}\right|}$$ 5

### 2.2 Jordan–Wigner Transformation

One approach to quantum computation of chemical problems is to utilize the second quantized formulation of quantum mechanics and convert the standard representation of fermionic quantities to the qubit representation. More specifically, we wish to find a mapping from “occupation number” (ON) vectors^50^ in Fock space to the qubit representation, in such a manner that the canonical (anti)­commutation relations of creation/annihilation operators acting on the ON vectors are preserved. This procedure is referred to most commonly as “encoding” or “mapping”.^2,3^ In this work, we utilize the Jordan-Wigner transformation (JWT)^51^ for mapping the fermionic operators to the qubit representation.

Assuming only a single particle type, the fermionic canonical anticommutation relations are

$$\{a_{p},a_{q}^{†}\}=\delta_{pq},\{a_{p}^{†},a_{q}^{†}\}=0,\{a_{p},a_{q}\}=0$$ 6

where *p*, *q* ∈ *S*_γ_. The form of the JWT is

$$a_{p}^{†}=(\underset{q>p}{⊗}Z_{q})⊗\sigma_{p}^{‐}⊗(\underset{r<p}{⊗}I_{r})$$ 7

$$a_{p}=\left(\underset{q>p}{⊗}Z_{q}\right)⊗\sigma_{p}^{+}⊗\left(\underset{r<p}{⊗}I_{r}\right)$$ 8

where *X*_*p*_, *Y*_*p*_, and *Z*_*p*_ are the Pauli matrices, *I* is the identity matrix, and $\sigma_{p}^{\pm }=\frac{1}{2}(X_{p}\pm iY_{p})$ . The mapping in eqs 7 and 8 needs to be altered for the case of multiple particle types. The canonical anticommutation relations are applicable to particles of the same type, but operators commute *across* particle types. Therefore, for different particle types, we have

$$[a_{p},a_{q}^{†}]=0,[a_{p}^{†},a_{q}^{†}]=0,[a_{p},a_{q}]=0$$ 9

where *p* ∈ *S*_γ_, *q* ∈ *S*_η_, γ≠η. For a multicomponent system with many types of fermionic particles, their respective creation/annihilation operators will all mutually commute. The JWT for a system comprised of a number of different particle types may then be written

$$a_{p}^{†}=\left(\underset{s}{⊗}I_{s}\right)⊗\left(\underset{q>p}{⊗}Z_{q}\right)⊗\sigma_{p}^{-}⊗\left(\underset{r<p}{⊗}I_{r}\right)⊗\left(\underset{u}{⊗}I_{u}\right)$$ 10

$$a_{p}=\left(\underset{s}{⊗}I_{s}\right)⊗\left(\underset{q>p}{⊗}Z_{q}\right)⊗\sigma_{p}^{+}⊗\left(\underset{r<p}{⊗}I_{r}\right)⊗\left(\underset{u}{⊗}I_{u}\right)$$ 11

where *p*, *q*, *r* ∈ *S*_γ_, *s* ∈ ∪_η>γ_ *S*_η_, and *u* ∈ ∪_η<γ_ *S*_η_. The form of eqs 10 and 11 is generally applicable to the case of a composite system of many types of fermions, where there could be many particles of each type. However, now consider a system where all individual particles are distinguishable. In that case, eqs 10 and 11 may be further simplified by the replacement of *Z*_*q*_ with *I*_*q*_. This is because there are no phases to be encoded by the operators to ensure proper spin-statistics for a single particle. Consequently, in the case of CNEO for all γ≠e, eqs 10 and 11 may be used, or more conveniently, the alternative version replacing *Z* with *I* may be used.

### 2.3 Constrained Nuclear-Electronic Orbital Full Configuration Interaction

We expand the total wave function |Ψ⟩ in an orthonormal multicomponent basis $\left|\psi_{i}\right\rangle$ spanning the composite Hilbert space H . Let

$$H=\underset{\gamma }{⊗}H^{\gamma }$$ 12

where the elements of the basis of each subspace $H^{\gamma }$ are represented by state vectors $\left|\Phi_{j}^{\gamma }\right\rangle$ . Then we may represent the composite multicomponent state according to

$$\left|\Psi \right\rangle =\underset{i}{\sum }c_{i}\left|\psi_{i}\right\rangle$$ 13

where

$$\left|\psi_{i}\right\rangle \in \left\{\left|\Phi_{j}^{\gamma }\right\rangle ⊗\left|\Phi_{k}^{\eta }\right\rangle ⊗\cdots ⊗\left|\Phi_{l}^{\kappa }\right\rangle \right\}$$ 14

Writing the Lagrangian for the CNEO energy minimization,^32^ we have

L=⟨Ψ|H^|Ψ⟩−E(⟨Ψ|Ψ⟩−1)+∑γfγ·(⟨Ψ|R^γ−Oγ|Ψ⟩)(15)=∑ijci*cjHij−E(∑i|ci|2−1)+∑γ∑ijfγ·(ci*cj⟨ψi|R^γ−Oγ|ψj⟩)(16)

where the vector ***f*** ^γ^ and scalar *E* are Lagrange multipliers, and ***O*** ^γ^ is the classical position for particle γ. The operator that describes the position shift relative to ***O*** ^γ^ may be represented according to

$${\hat{R}}_{\alpha }^{\gamma }-O_{\alpha }^{\gamma }=\sum_{pq\in S_{\gamma }}{\overset{-}{r}}_{\alpha ,pq}^{\gamma }a_{p}^{†}a_{q}$$ 17

with

$${\overset{-}{r}}_{\alpha ,pq}^{\gamma }=\langle \phi_{p}|{\hat{r}}_{\alpha }^{\gamma }-O_{\alpha }^{\gamma }|\phi_{q}\rangle =\int dx^{\gamma }\phi_{p}^{*}(x^{\gamma })(r_{\alpha }^{\gamma }-O_{\alpha }^{\gamma })\phi_{q}(x^{\gamma })$$ 18

where α is a Cartesian component α ∈ {*x*, *y*, *z*}.

Differentiating with respect to *c*_k_^*^ and setting equal to zero we obtain

$$\frac{\partial L}{\partial c_{k}^{*}}=\sum_{j}H_{kj}c_{j}-Ec_{k}+\sum_{\gamma }\sum_{j}f^{\gamma }\cdot \langle \psi_{k}|{\hat{R}}^{\gamma }-O^{\gamma }|\psi_{j}\rangle c_{j}=0$$ 19

which when cast as a matrix equation reads

$$\left[H+\sum_{\gamma }f^{\gamma }\cdot {\overset{-}{R}}^{\gamma }\right]C=CE$$ 20

where ${\overset{-}{R}}_{kj}^{\gamma }=\langle \psi_{k}|{\hat{R}}^{\gamma }-O^{\gamma }|\psi_{j}\rangle$ is a vector of matrices. The quantity in brackets in eq 20 represents a kind of effective Hamiltonian matrix, which is equivalent to the standard Hamiltonian matrix plus the constraint terms. Upon transformation of the creation and annihilation operators to the qubit representation, **H** and ${\overset{-}{R}}^{\gamma }$ can be constructed using eqs 1 – [Disp-formula eq5] and eqs 17 – [Disp-formula eq18], respectively.

### 2.4 Constrained Nuclear-Electronic Orbital Unitary Coupled Cluster

Quantum algorithms for multicomponent unitary coupled-cluster have been developed previously.^15^ The CNEO method differs significantly from previous work in that (1) the cluster amplitudes are optimized subject to the quantum nuclear expectation value constraint, resulting in a well-defined surface, and (2) the distinguishable particle approximation is used, so the form of the Hamiltonian operator in eq 1 as well as the forms the cluster operators are different relative to the indistinguishable case.

The multicomponent unitary coupled-cluster^15–17^ wave function ansatz is written as

$$\left|\Psi \rangle =e^{\hat{T}-{\hat{T}}^{†}}|\psi_{0}\right\rangle$$ 21

where |ψ_0_⟩ is a multicomponent Hartree–Fock reference

$$\left|\psi_{0}\right\rangle =\underset{\gamma }{⊗}\left|\Phi_{0}^{\gamma }\right\rangle$$ 22

Using similar notation to that of refs 16 and 17, the cluster operator is expanded according to

$$\hat{T}=\sum_{o_{\gamma },o_{\eta },\cdots }{\hat{T}}^{\left(o_{\gamma },o_{\eta },\cdots \right)}$$ 23

where *o*_γ_, *o*_η_, ··· represent the order of the particle-hole excitations for the respective particle types. Because we assume that all particles that are not electrons are distinguishable, the excitation rank for those particles can be at most one. Additionally, we restrict the cluster operator to contain only particle-conserving excitations. The summand of a generic excitation operator is then given according to

$$t_{ij\cdots kl\cdots }^{ab\cdots cd\cdots }\underset{e}{\underset{︸}{a_{a}^{†}a_{i}a_{b}^{†}a_{j}\cdots \;}}\underset{\gamma }{\underset{︸}{a_{c}^{†}a_{k}\;}}\underset{\eta }{\underset{︸}{a_{d}^{†}a_{l}\;}}\cdots$$ 24

where *i*, *j* ∈ *S*_e_^o^, *a*, *b* ∈ *S*_e_^v^, *k* ∈ *S*_γ_^o^, *c* ∈ *S*_γ_^v^, *l* ∈ *S*_η_^o^, *d* ∈ *S*_η_^v^, and *t*_*ij...kl...*_^*ab...cd...*^ is a generic cluster amplitude. It is understood that γ, η≠e.

We wish to minimize the energy with respect to the cluster amplitudes, subject to the constraint on the expectation values of the quantum nuclei. Here, we modify the constrained optimization problem into a restrained optimization problem by applying a penalty term to the energy functional according to

$$E=\min_{t}\left[\langle \Psi |\hat{H}|\Psi \rangle +\mu \sum_{\gamma }\langle \Psi |{\hat{R}}^{\gamma }-O^{\gamma }|\Psi \rangle^{2}\right]$$ 25

where μ is some fixed large number. Once the cluster amplitudes are obtained from the minimization in eq 25, they are used to evaluate the energy *E* = ⟨Ψ|*Ĥ*|Ψ⟩. This restraint procedure does not rigorously satisfy the constraint, but with proper choice of μ can give accurate results. Note that restraint procedures of this form have been previously explored for quantum algorithms.^52^ For practical purposes, we may truncate the cluster operator at various orders, with one popular choice of truncation being at double excitations. This results in the CNEO-UCC singles and doubles (CNEO-UCCSD) method. Because there are potentially many particle types, truncating the cluster operator at doubles is defined as including only those operators *T̂* ^(*o*_γ_,*o*_η_,···)^ in the sum in eq 23 for which ∑_γ_ *o*_γ_ ≤ 2.

For the specific case of diatomic molecules, if we let the first position in the superscript correspond to electrons and the next two correspond to the distinguishable nuclei n_1_ and n_2_ (*T̂* ^(e,n_1_,n_2_)^), then the CNEO singles and doubles cluster operator *T̂*_SD_ can be written

$${\hat{T}}_{\mathrm{SD}}={\hat{T}}^{\left(0,0,1\right)}+{\hat{T}}^{\left(0,1,0\right)}+{\hat{T}}^{\left(1,0,0\right)}+{\hat{T}}^{\left(0,1,1\right)}+{\hat{T}}^{\left(1,0,1\right)}+{\hat{T}}^{\left(1,1,0\right)}+{\hat{T}}^{\left(2,0,0\right)}$$ 26

In contrast to the conventional electronic case, note that CNEO-UCCSD is not exact for the hydrogen isotopologues studied herein, with the missing terms in the cluster operator in eq 26 being due to triple (T) and quadruple (Q) excitations. After transformation of the creation and annihilation operators to the qubit basis, the CNEO-UCCSD amplitudes can be solved by direct minimization of the modified energy functional in eq 25, where the states |Ψ⟩ are constructed according to eq 21.

### 2.5 von Neumann Entropy

The von Neumann entropy (VNE) is a measure of entanglement between quantum subsystems of a composite system. It has been used previously^16^ in the quantum computing implementation of the NEO method to measure electron–proton entanglement. In the case of CNEO, with the distinguishable particle assumption, the number of quantum subsystems equals the number of quantum nuclei plus one (electronic subsystem). For example, the diatomic species studied herein constitute tripartite quantum systems: One electronic subsystem and two separate quantum nuclear subsystems.

The VNE is generally defined as

$$S(\hat{\rho })=-\mathrm{Tr}[\hat{\rho }\ln(\hat{\rho })]$$ 27

In the case of a composite quantum system where A is a subsystem, we represent the VNE of the subsystem A according to *S*(A) ≡ *S*(ρ̂^A^), where ρ̂^A^ is the reduced density operator formed by tracing out all subsystems not equal to A. The VNE is always positive, and is zero if and only if the state in question is pure.^53^ Another useful quantity is the mutual information

I(A:B)=S(A)+S(B)−S(AB) 28

which is a measure of the correlation between subsystems A and B. It can be viewed as the distance of the state ρ̂^AB^ from the uncorrelated state ρ̂^A^ ⊗ ρ̂^B^,^54^ and returns zero if ρ̂^AB^ = ρ̂^A^ ⊗ ρ̂^B^ due to the property *S*(ρ̂^A^ ⊗ ρ̂^B^) = *S*(ρ̂^A^) + *S*(ρ̂^B^).^53^ We note also that for pure states, the subsystem entropies of any bipartition of the composite system are equivalent. For example, in a tripartite system, the bipartitions $H^{A}⊗H^{\mathrm{BC}}$ , $H^{B}⊗H^{\mathrm{CA}}$ , and $H^{C}⊗H^{\mathrm{AB}}$ have the conditions *S*(A) = *S*(BC), *S*(B) = *S*(CA), and *S*(C) = *S*(AB), respectively. This result is a consequence of the Schmidt decomposition.^53^ Finally, we note that for a tripartite quantum system, there is an important inequality condition that the entropy must satisfy, known as strong subadditivity^55–59^

S(ABC)+S(B)≤S(AB)+S(BC) 29

## 3 Results

### 3.1 Computational Details

We have calculated potential energy surfaces, optimized geometries, harmonic vibrational frequencies, and von Neumann entropies for the H_2_, HD, and D_2_ molecules using CNEO-FCI and CNEO-UCCSD. Calculations were performed using a locally modified version of the PySCF software program.^60^ All simulations were performed on a purely classical machine, which represents an exact, noise-free state-vector simulation of the VQE. In these systems, both nuclei are treated as distinguishable quantum particles. For all calculations, we employed the 6–31G electronic basis set and a custom nuclear basis set consisting of two *s*-type functions and a single Cartesian *p*-type function oriented along the internuclear axis. This results in a total of 14 qubits (8 for the electrons, and 3 for each quantum nucleus).

The nuclear basis exponent values were 20.17583 and 29.29266 for the *s*-type functions and 22.19227 for the *p*-type function. These values were taken from Table 1 of ref 22., with the *p*-type exponent being an average of the reported values. The exponents of the deuteron were scaled according to $\sqrt{m_{D}/m_{H}}$ . The nuclear basis function centers are placed at the target position for the expectation value in every calculation. The CNEO-UCCSD and CNEO-FCI results used CNEO-HF reference orbitals with the maximum norm of the orbital gradient set to 10^–7^.

The CNEO-FCI and CNEO-UCCSD methods both use SciPy^61^ for parameter optimization. For CNEO-FCI, root finding functionality is used. The variables of interest are the Lagrange multipliers ***f*** ^γ^. The effective Hamiltonian matrix in eq 20 is constructed using the Jordan-Wigner transformation and CNEO-HF orbitals, and subsequently diagonalized. The state of interest is then used to check if the constraint condition has been satisfied. If not, the Lagrange multipliers are updated and the procedure continues until the roots are located.

The CNEO-UCCSD amplitudes are solved according to the minimization in eq 25 using the Broyden-Fletcher-Goldfarb-Shanno (BFGS) algorithm with μ = 10^4^. This value of μ was found to yield an error in the expectation value position of the quantum nuclei on the order of 10^–6^ bohr in the vicinity of the equilibrium geometry. The error in energy was found to be on the order of ≈10^–4^ to 10^–6^ hartree in the case of small bond distances, while the errors are much smaller (≈10^–7^ hartree or less) for bond distances near equilibrium and beyond. While the value of μ can be increased to achieve more accurate results in individual cases, this can lead to difficult convergence behavior in general, depending on the system and bond length. As such, the current choice of μ represents a good balance between accuracy and convergence. For a more complete analysis of the errors associated with the CNEO-UCCSD energies and entropies, see Tables S1–S3 in the Supporting Information.

### 3.2 Surfaces, Geometries, and Vibrational Frequencies

The CNEO-UCCSD PESs for the H_2_ HD, and D_2_ molecules are shown in Figure 1, along with the conventional electronic PES for the case where both nuclei are treated classically. All curves have been shifted such that zero energy corresponds to the dissociation limit. The electronic PES has the deepest well, corresponding to the largest binding energy. This is because it does not contain zero-point energy (ZPE) contributions from any quantum nuclei. Conversely, the binding energies of all molecular species calculated using CNEO are smaller because they inherently contain ZPE.^33^ Among the molecules, H_2_ has the smallest binding energy while D_2_ has the largest. This is due to the differing masses of the nuclei in question, and thus differing amounts of ZPE. It is expected that as nuclear mass increases the results should approach the case in which the nuclei are treated as classical point charges. These observations are consistent with past CNEO–DFT results.^33^

**Figure 1:**
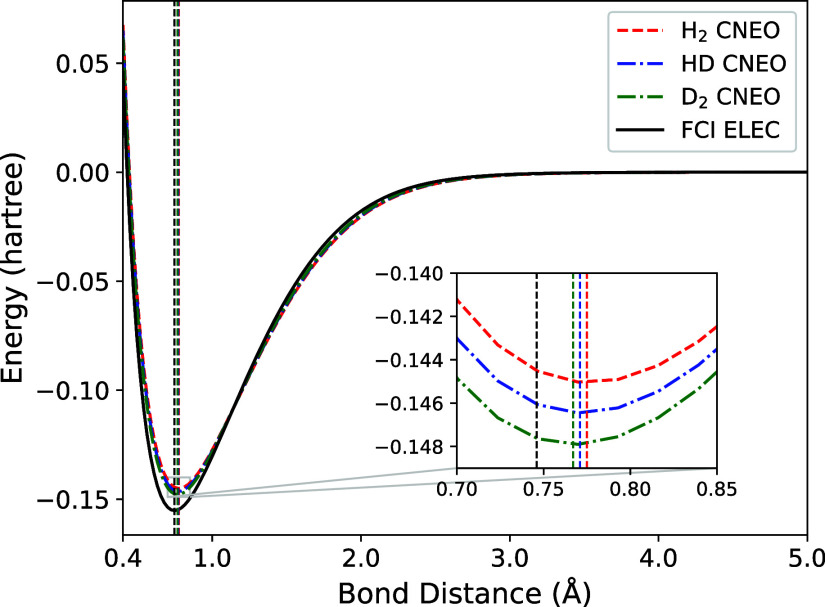
Potential energy surfaces calculated using CNEO-UCCSD as a function of bond distance for H_2_, HD, and D_2_. Conventional electronic FCI results are shown by the solid black line. For two electron systems, conventional CCSD is equivalent to FCI and so only conventional FCI is plotted. The inset shows a magnification of the area around equilibrium for each surface. Vertical dashed lines indicate equilibrium bond lengths for each molecule.

The differences in equilibrium bond length for the different isotopologues are evident from the inset in Figure 1, and reflect the geometric isotope effect. The values of the optimized geometries are reported in Table 1. These results constitute a proof of principle, and demonstrate that equilibrium geometries can be calculated in the CNEO paradigm using quantum algorithms. Because of the basis set limitations, quantitative accuracy is not expected, but qualitative trends can still be observed. It is important to note that the reported experimental equilibrium bond lengths (*R*_e_) and those calculated using conventional electronic structure methods are typically associated with the minimum of the Born–Oppenheimer potential energy surface and are independent of nuclear mass. In contrast, the CNEO-optimized geometries correspond to the expectation values of quantum nuclear positions and are more aligned with *R*_0_, the vibrationally averaged bond lengths. Since geometric isotope effects arise from nuclear quantum delocalization, they are manifested in *R*_0_ rather than *R*_e_, and are naturally captured within the CNEO framework.

**Table 1:** Bond Lengths of H_2_, HD, and D_2_ (Å)

molecule	experiment^a^	CNEO-FCI^b^	CNEO-UCCSD	CNEO-HF	FCI	HF
H_2_	0.742	0.777	0.777	0.771	0.746	0.730
HD	0.742	0.772	0.772	0.765	0.746	0.730
D_2_	0.742	0.767	0.767	0.759	0.746	0.730

a *R*_e_ values taken from ref 62.

b Due to small basis set, results cannot be quantitatively compared to the experiment.

The geometries reported in Table 1 show that the CNEO results tend to increase bond distances relative to their standard electronic structure counterparts, consistent with past CNEO–DFT studies.^33^ This can be understood by the fact that the nuclei in CNEO calculations are delocalized and the potential is asymmetric. The CNEO-FCI and CNEO-UCCSD geometries are the same to within the reported digits, which is unsurprising given the accuracy of CNEO-UCCSD relative to CNEO-FCI (see Figure 3). The CNEO-FCI and CNEO-UCCSD geometries are elongated relative to CNEO-HF, a similar trend to the conventional FCI and HF results.

The differences between the CNEO-FCI and CNEO-UCCSD energies as a function of bond distance are shown in Figure 2. Starting from short bond distances, the magnitude of the energy difference begins decreasing, and reaches a minimum near the equilibrium geometry. The magnitude of the difference then increases until ≈2 Å, where it begins to level off. In the separate atom limit, the energy difference approaches a constant value. We note that the energy difference is always negative, which should be the case given that both methods are variational and CNEO-FCI has full correlation, whereas CNEO-UCCSD misses the correlation due to T and Q excitations. The relative magnitude of the differences are also distinct based on mass, with the heavier species having less difference in energy. For all molecules, the magnitude of the energy differences are small, with most falling between 0.1 to 0.8 millihartree. Examining Figure 3, we see that this corresponds to CNEO-UCCSD recovering >99% of the correlation energy for most bond distances.

**Figure 2:**
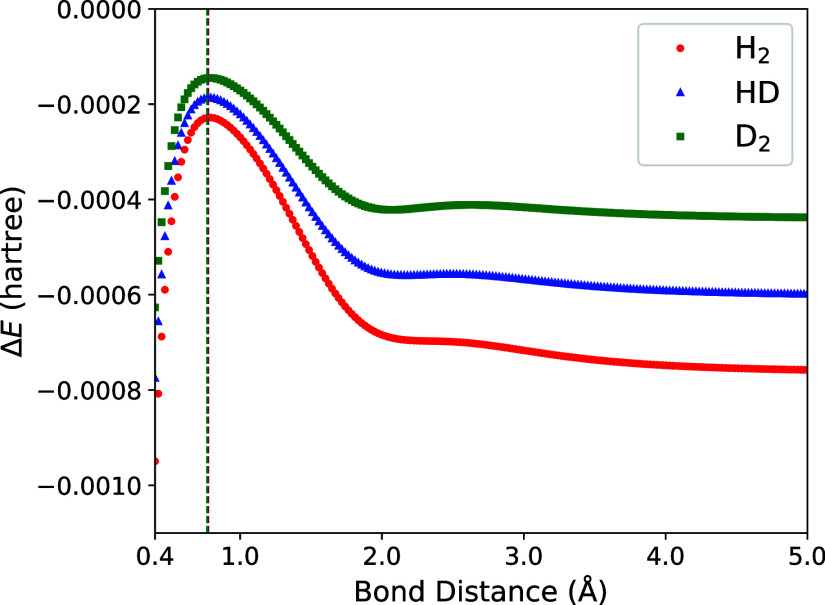
Difference in ground state energy *E* for CNEO-FCI and CNEO-UCCSD as a function of bond distance. The UCCSD energy is subtracted from the FCI energy. Vertical dashed lines indicate equilibrium bond lengths for each molecule.

**Figure 3:**
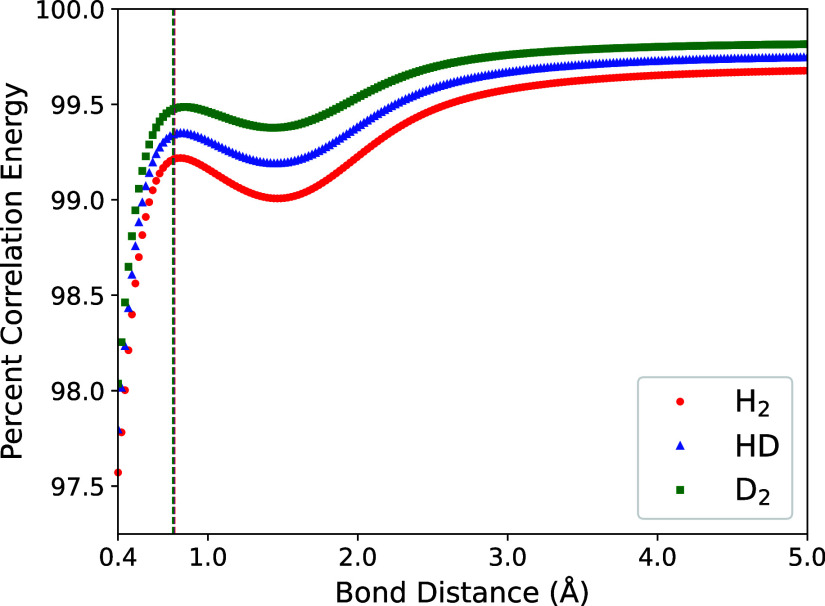
Percentage correlation energy recovered by CNEO-UCCSD as a function of bond distance for H_2_, HD, and D_2_. Vertical dashed lines indicate equilibrium bond lengths for each molecule.

The good performance of CNEO-UCCSD for the simple H_2_ isotopologues studied here is expected, as electron–electron and nuclear–nuclear correlations are fully captured through the inclusion of single and double (S and D) excitations. Only a portion of the electron–nuclear correlation that is associated with triple and quadruple (T and Q) excitations is missing. For larger systems, where CNEO-UCCSD may no longer capture all electron–electron and nuclear–nuclear correlations, the percentage of recovered correlation energy may decrease. Nonetheless, the method is still expected to maintain a relatively high level of accuracy, given the well-established performance of conventional UCCSD in describing electron–electron correlation.^63–66^

Given that CNEO allows for the calculation of well-defined surfaces in terms of the expectation values of nuclear densities, harmonic vibrational frequencies can be obtained from these surfaces. Second derivatives were calculated at the equilibrium geometry of each species numerically using a five-point stencil and a step size of 10^–3^ Å. Frequencies are then calculated according to $\omega =\sqrt{k/\mu_{m}}$ , where μ_m_ is the reduced mass and *k* is the second derivative in the harmonic approximation. Results are shown in Table 2. For the conventional electronic structure case, the FCI results are lower than the HF results by ≈200–300 cm^–1^ depending on the species. This trend is similar for the CNEO results, although the range is smaller, with CNEO-FCI being lower than CNEO-HF by ≈150–200 cm^–1^ depending on the species. In all cases, the CNEO results are lower than the corresponding conventional electronic structure results, due to nuclear quantum effects. The CNEO-HF results are closest to the experimental results, but this is again a consequence of the small basis set size. Finally, the CNEO-UCCSD results are nearly identical to the CNEO-FCI results, being within 2 to 4 cm^–1^ in all cases. This is expected given the accuracy of CNEO-UCCSD.

**Table 2:** Harmonic Vibrational Frequencies of H_2_, HD, and D_2_ (cm^–1^)

molecule	experiment^a^	CNEO-FCI^b^	CNEO-UCCSD	CNEO-HF	FCI	HF
H_2_	4161	4019	4023	4214	4368	4647
HD	3632	3525	3528	3704	3783	4025
D_2_	2994	2915	2917	3070	3089	3287

a From the National Institute of Standards and Technology (NIST) websites.

b Due to small basis set, results cannot be quantitatively compared to the experiment.

### 3.3 von Neumann Entropy

The VNE is a measure of entanglement between quantum subsystems, making it an interesting quantity to examine. This is especially so using CNEO, for which both heteronuclear and homonuclear diatomic molecules constitute tripartite quantum systems. The difference between CNEO-FCI and CNEO-UCCSD entropy as a function of bond distance for *S*(e) is shown in Figure 4, where the argument “e” represents the electronic reduced density operator (see eq 27).

**Figure 4:**
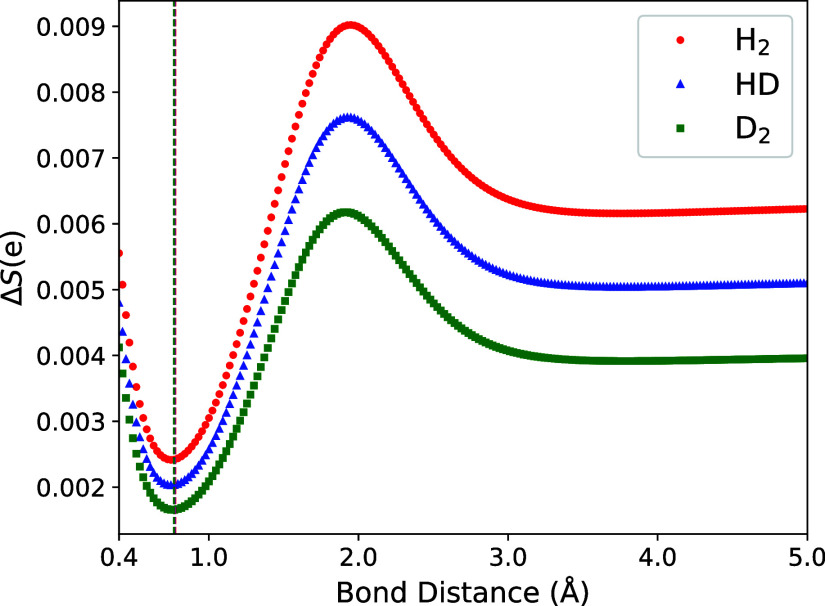
Difference in von Neumann entropy *S*(e) for CNEO-FCI and CNEO-UCCSD as a function of bond distance. The UCCSD entropy is subtracted from the FCI entropy. Vertical dashed lines indicate equilibrium bond lengths for each molecule.

We see that the trends in VNE differences are qualitatively similar to the energy differences plotted in Figure 2. There is a minimum present near equilibrium, a maximum present around ≈2 Å, and the curves approach a constant value in the separate atom limit. Recall that the CNEO-UCCSD method is missing T and Q contributions, which are purely electron–nuclear excitations. Because *S*(e) is a measure of the entanglement of the electrons with the quantum nuclei, it is therefore reasonable that the differences plotted in Figure 4 would show qualitatively similar trends to those of Figure 2. Moreover, the entropy difference is always positive, as we should expect given that CNEO-FCI includes more correlation than CNEO-UCCSD. Finally, for the three molecules studied, the magnitude of the difference for H_2_ is always largest while for D_2_ it is always smallest. This trend makes sense because as mass increases the problem should approach the conventional case of fixed point charges, where there is no difference between FCI and UCCSD for two electrons.

The subsystem entropies *S*(e), *S*(n_1_), and *S*(n_2_) are shown in Figure 5, calculated using CNEO-FCI as a function of bond distance for H_2_ (panel (a)), HD (panel (b)), and D_2_ (panel (c)). The arguments n_1_ and n_2_ are generic labels that represent the reduced density operators of the quantum nuclei for each diatomic species. We note that the behavior of the entropy as a function of bond distance is expected to change qualitatively with basis set size at short and intermediate distances. Therefore, we will be concerned primarily with an examination of the dissociation limit, where we should expect certain qualitative trends to be maintained across most basis sets.

**Figure 5:**
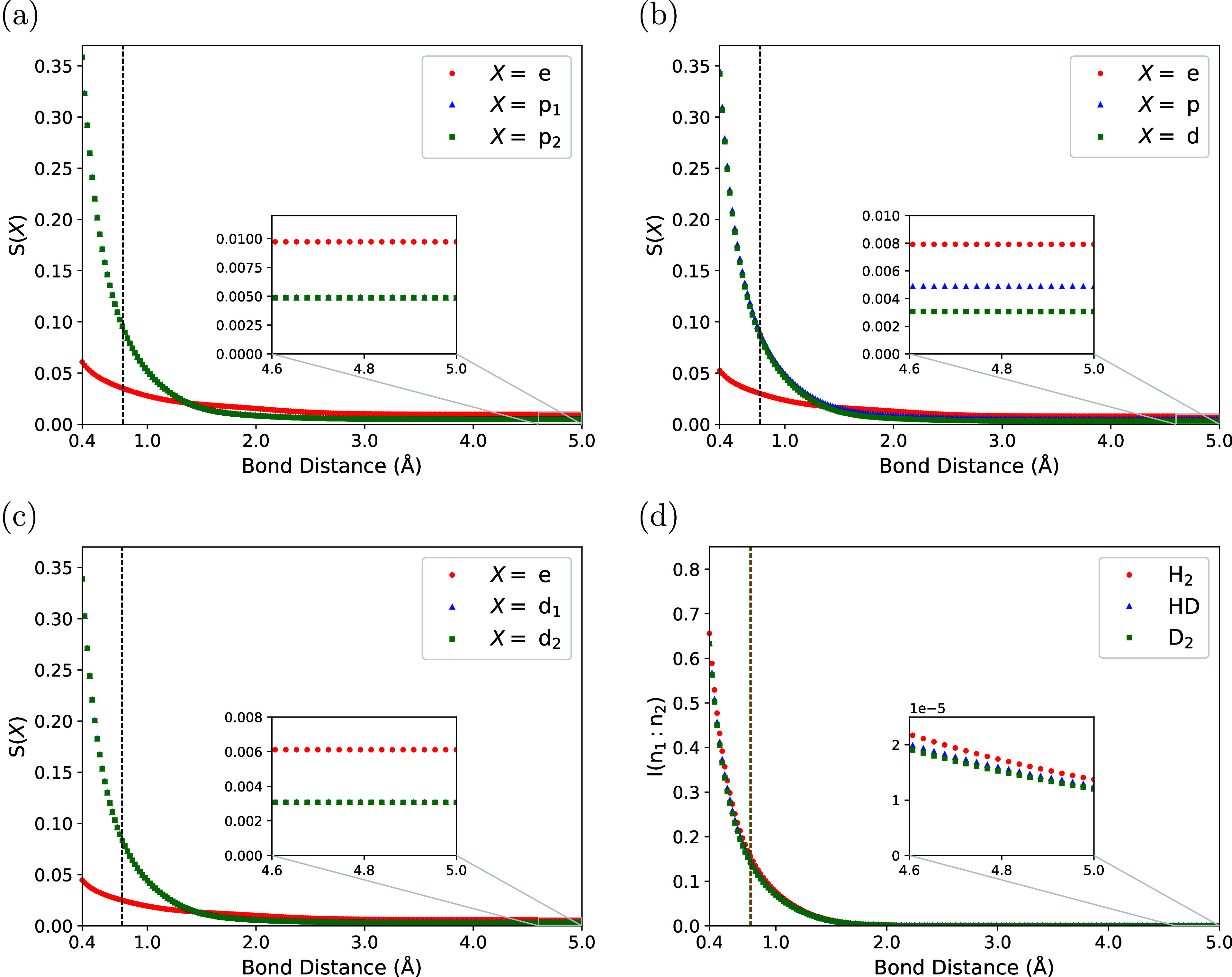
Subystem von Neumann entropy for H_2_ (panel (a)), HD (panel (b)), and D_2_ (panel (c)) calculated using CNEO-FCI as a function of bond distance. The mutual information between the quantum nuclei for each diatomic molecule is shown in panel (d). The inset in each panel is a magnification of the region corresponding to large internuclear separation. Vertical dashed lines in each panel correspond to equilibrium bond distances. The quantum particle labels in panels (a–c) are “e” for electron, “p” for proton, and “d” for deuteron.

In Figure 5, the entropies for all molecules are observed to decrease monotonically as a function of increasing bond distance, and approach constant values at large internuclear separation. Comparing entropy values for different species at long bond distances, we see that the *S*(e) values decrease with increasing total nuclear mass. This is because electron–nuclear entanglement for isolated atoms should decrease with increasing nuclear mass. The inset in each panel in Figure 5 displays a magnification of the behavior at longer bond distances. Here it is observed that *S*(e) ≈ *S*(n_1_) + *S*(n_2_) for all molecules when the internuclear separation is large. This is an indication that strong subadditivity holds with equality in this regime. To see this, recall eq 29. If we identify B with the electronic subsystem, A with the nuclear subsystem n_1_, and C with the nuclear subsystem n_2_, then the strong subadditivity condition can be written as

$$S(en_{1}n_{2})+S(e)\leq S(n_{1}e)+S(en_{2})$$ 30

Using the equivalence of entropies of bipartitions (see Section 2.5), and the fact that the VNE of a pure state is zero, the strong subadditivity condition may be equivalently represented as

$$S(e)\leq S(n_{1})+S(n_{2})$$ 31

In the separate atom limit it is the case that the electrons may be locally entangled with their respective quantum nuclei, resulting in a nonzero VNE for all subsystems involved, yet the nuclei themselves are uncorrelated, i.e., ρ̂^n_1_n_2_^ = ρ̂^n_1_^ ⊗ ρ̂^n_2_^ (see the Supporting Information for a more rigorous mathematical proof). Because the mutual information is zero for an uncorrelated state, we have

$$I(n_{1}:n_{2})=S(n_{1})+S(n_{2})-S(n_{1}n_{2})=0$$ 32

and therefore

$$S(e)=S(n_{1}n_{2})=S(n_{1})+S(n_{2})$$ 33

Comparing eqs 31 and 33, we see that the condition of strong subadditivity between the electrons and the quantum nuclei holds with equality, and does so nontrivially because *S*(γ) > 0 ∀ γ.

Calculation of the quantum nuclear mutual information according to eq 28 confirms this result, as shown in Figure 5d. At long bond distances, the inset shows that the mutual information is greater for the lighter species as expected (H_2_ > HD > D_2_), but the overall magnitude of the mutual information is quite low. Looking at the values of the subsystem VNEs, the mutual information at large bond distances is ≈100 times lower than any individual VNE, indicating that the quantum nuclei have relatively little correlation, consistent with the proof that the nuclear mutual information should tend to zero in the separate atom limit. Note that this result is for the case of distinguishable nuclei, where nuclear spin correlation is not considered.

## 4 Conclusions

In this work, we have presented the CNEO-FCI and CNEO-UCC methods for quantum computation. This represents a first step toward the development of correlated wave function methods for CNEO. The CNEO-UCCSD method was solved using the VQE algorithm to demonstrate its applicability for quantum computation of molecular properties. This is achieved through the Jordan-Wigner transformation of the electronic and nuclear creation and annihilation operators. Minimization is performed subject to a penalty term in the energy functional that serves to restrain the expectation value of the quantum nuclei to some predefined target, facilitated by the second-quantized representation of the position operator.

The CNEO-FCI and CNEO-UCCSD methods were used to calculated potential energy surfaces, equilibrium geometries, harmonic vibrational frequencies, and von Neumann entropies for the hydrogen isotopologues H_2_, HD, and D_2_. The CNEO-UCCSD energies, geometries, and harmonic frequencies were found to be in excellent agreement with CNEO-FCI. For the systems studied, CNEO-UCCSD recovers >99% of the correlation energy, yields the same geometries to the reported digits, and is within 2 to 4 cm^–1^ of the CNEO-FCI vibrational frequencies, thus demonstrating a high level of accuracy. Due to the incorporation of nuclear quantum effects, the CNEO methods were found to elongate equilibrium bond lengths relative to conventional FCI, as well as lower the vibrational frequencies. These observations are consistent with previous CNEO studies using DFT.

While the difference in the CNEO-FCI and CNEO-UCCSD energy is small, it is qualitatively similar to differences in the von Neumann entropies *S*(e) of the two methods. It was observed that near equilibrium both the energy and entropy differences tend to be low in magnitude, increase as the bond is stretched, and finally approach constant values in the dissociation limit. This observed connection between the entropy difference and the energy difference is interesting because of the interpretation of the entropy as a measure of entanglement between quantum subsystems.

Finally, CNEO-FCI entropies were examined as a function of bond distance, and it was found that in the separate atom limit the mutual information between the quantum nuclei tends to zero, and the strong subadditivity condition between the electronic subsystem and the quantum nuclear subsystems approaches equality nontrivially. This work lays the foundation for the implementation of CNEO methods on quantum devices, as well as research into electron–nuclear interactions from a quantum information theory perspective using CNEO.

## Supplementary Material



## Data Availability

The code developed and used in this work is publicly available at https://github.com/theorychemyang/pyscf.

## Notes

Additional details and calculations: Mathematical analysis of quantum nuclear von Neumann entropy in the separate atom limit; and error analysis of the CNEO-UCCSD restraint procedure (PDF)
