# Supplementary material for: Constrained Nuclear-Electronic Orbital Theory for Quantum Computation
Source: J Chem Theory Comput. 2025 Aug 12;21(16):7845–54. doi: 10.1021/acs.jctc.5c00815 (PMC12503367; doi:10.1021/acs.jctc.5c00815)
Supplement: Supplementary file 1 [file ct5c00815_si_001.pdf]

# Supporting Information: Constrained Nuclear-Electronic Orbital Theory for Quantum Computation

Tanner Culpitt,[*,†] Zehua Chen,[†] Fabijan Pavošević,[‡] and Yang Yang[*,†]

†*Theoretical Chemistry Institute and Department of Chemistry, University of Wisconsin-Madison, 1101 University Ave, Madison, Wisconsin 53706 USA*

‡*Algorithmiq Ltd., Kanavakatu 3C, FI-00160 Helsinki, Finland*

E-mail: tculpitt@wisc.edu; yyang222@wisc.edu

## S1 Nuclear-nuclear mutual information in the separate atom limit

We consider a diatomic molecular species comprised of two electrons and two distinguishable quantum nuclei in a singlet electronic state. Let $|\text{ep}\rangle$ represent the state of an atomic species with electronic spin $\uparrow$ and $|\bar{\text{e}}\text{p}\rangle$ represent the state of an atomic species with electronic spin $\downarrow$, where the labels e and p denote the electron and quantum nucleus, respectively. In the atomic state $|\text{ep}\rangle$, the electron-nuclear system is entangled, and we may write

$$|\text{ep}\rangle = \sum_{ij} C_{ij} \, |\text{e}_b^i\rangle \, |\text{p}_b^j\rangle \, , \tag{S1}$$

where $C_{ij}$ are expansion coefficients, $|\text{e}_b^i\rangle$ are basis states of the electronic Hilbert space, and $|\text{p}_b^j\rangle$ are basis states of the nuclear Hilbert space. Note that because $|\text{ep}\rangle$ and $|\bar{\text{e}}\text{p}\rangle$ differ

only by the spin of the electron, the expansion coefficients for these states are identical. The Schmidt decomposition of Eq. (S1) gives

$$|\mathrm{ep}\rangle = \sum_m \lambda_m \, |\mathrm{e}^m_{\mathrm{p}s}\rangle \, |\mathrm{p}^m_s\rangle \,, \tag{S2}$$

where $\lambda_m$ are the Schmidt coefficients and $|\mathrm{e}^m_{\mathrm{p}s}\rangle$ and $|\mathrm{p}^m_s\rangle$ are the electronic and nuclear Schmidt vectors, respectively. Again, note that the Schmidt coefficients of the states $|\mathrm{ep}\rangle$ and $|\bar{\mathrm{e}}\mathrm{p}\rangle$ will be identical. The reduced density operators for the electronic and nuclear subsystems are then given by

$$\hat{\rho}^{\mathrm{e}} = \mathrm{Tr}_{\mathrm{p}}\big[\, |\mathrm{ep}\rangle \, \langle \mathrm{ep}| \,\big] = \sum_m \lambda_m^2 \, |\mathrm{e}^m_{\mathrm{p}s}\rangle \, \langle \mathrm{e}^m_{\mathrm{p}s}| \,, \tag{S3}$$

$$\hat{\rho}^{\mathrm{p}} = \mathrm{Tr}_{\mathrm{e}}\big[\, |\mathrm{ep}\rangle \, \langle \mathrm{ep}| \,\big] = \sum_m \lambda_m^2 \, |\mathrm{p}^m_s\rangle \, \langle \mathrm{p}^m_s| \,. \tag{S4}$$

Now, the total state for an electronic singlet of a diatomic species comprised of distinguishable quantum nuclei p and d in the separate atom limit may be written

$$|\Psi\rangle = \frac{1}{\sqrt{2}}(|\mathrm{ep}\rangle \, |\bar{\mathrm{e}}\mathrm{d}\rangle - |\bar{\mathrm{e}}\mathrm{p}\rangle \, |\mathrm{ed}\rangle). \tag{S5}$$

Using the Schmidt decomposition we obtain

$$|\Psi\rangle = \frac{1}{\sqrt{2}} \sum_{mn} \lambda_m \mu_n \big( \, |\mathrm{e}^m_{\mathrm{p}s}\rangle \, |\bar{\mathrm{e}}^n_{\mathrm{d}s}\rangle - |\bar{\mathrm{e}}^m_{\mathrm{p}s}\rangle \, |\mathrm{e}^n_{\mathrm{d}s}\rangle \, \big) \, |\mathrm{p}^m_s\rangle \, |\mathrm{d}^n_s\rangle \,. \tag{S6}$$

The density operator for this state is then

$$\begin{aligned}\hat{\rho} &= |\Psi\rangle \, \langle \Psi| \\ &= \frac{1}{2} \sum_{mnm'n'} \lambda_m \lambda_{m'} \mu_n \mu_{n'} \big( \, |\mathrm{e}^m_{\mathrm{p}s}\rangle \, |\bar{\mathrm{e}}^n_{\mathrm{d}s}\rangle - |\bar{\mathrm{e}}^m_{\mathrm{p}s}\rangle \, |\mathrm{e}^n_{\mathrm{d}s}\rangle \, \big) \big( \, \langle \bar{\mathrm{e}}^{n'}_{\mathrm{d}s}| \, \langle \mathrm{e}^{m'}_{\mathrm{p}s}| - \langle \mathrm{e}^{n'}_{\mathrm{d}s}| \, \langle \bar{\mathrm{e}}^{m'}_{\mathrm{p}s}| \, \big) \, |\mathrm{p}^m_s\rangle \, |\mathrm{d}^n_s\rangle \, \langle \mathrm{d}^{n'}_s| \, \langle \mathrm{p}^{m'}_s| \,.\end{aligned} \tag{S7}$$

Tracing over the electronic subsystem yields

$$\mathrm{Tr}_{\mathrm{e}}\big[\hat{\rho}\big] = \hat{\rho}^{\mathrm{pd}} = \sum_{mn} \lambda_m^2 \mu_n^2 \left|\mathrm{p}_s^m\right\rangle \left|\mathrm{d}_s^n\right\rangle \left\langle \mathrm{d}_s^n\right| \left\langle \mathrm{p}_s^m\right| = \hat{\rho}^{\mathrm{p}} \otimes \hat{\rho}^{\mathrm{d}}, \tag{S8}$$

from which it follows that

$$I(\hat{\rho}^{\mathrm{p}} : \hat{\rho}^{\mathrm{d}}) = S(\hat{\rho}^{\mathrm{p}}) + S(\hat{\rho}^{\mathrm{d}}) - S(\hat{\rho}^{\mathrm{pd}}) = 0. \tag{S9}$$

# S2 Error due to the restraint procedure in UCC

As mentioned in the main text, CNEO-UCCSD employs a restraint procedure, which does not rigorously satisfy the constraint. There will be a finite deviation between the target expectation value and the one actually achieved by CNEO-UCCSD in practice. For our chosen value of $\mu$, we investigate how much error is due to the restraint procedure. In order to estimate the error, we have implemented the missing triple and quadruple excitations (UCCSDTQ) for the diatomic systems studied in this work. In principle, CNEO-UCCSDTQ should agree exactly with CNEO-FCI. Therefore, any difference in energy between the two methods can be reasonably attributed to the lack of a rigorously satisfied constraint. Technically, the minimization procedure itself also contains inherent errors, but for the purpose of this test we assume the solver errors to be negligible compared to the constraint deviation error.

We define the following energy differences and percentage errors that are reported in Tables. S1– S3 (to avoid long acronyms, the CNEO label is omitted but assumed, and all entropy values are electronic, $S \equiv S(\mathrm{e})$):

$$\Delta E_{\mathrm{UCCSDTQ}} = E_{\mathrm{FCI}} - E_{\mathrm{UCCSDTQ}}, \tag{S10}$$

$$\Delta E_{\text{UCCSD}} = E_{\text{FCI}} - E_{\text{UCCSD}}, \tag{S11}$$

$$\% \text{ Error } \Delta E_{\text{UCCSD}} = \frac{|\Delta E_{\text{UCCSDTQ}}|}{|\Delta E_{\text{UCCSD}}|} \times 100 \tag{S12}$$

$$\Delta S_{\text{UCCSDTQ}} = S_{\text{FCI}} - S_{\text{UCCSDTQ}}, \tag{S13}$$

$$\Delta S_{\text{UCCSD}} = S_{\text{FCI}} - S_{\text{UCCSD}}, \tag{S14}$$

$$\% \text{ Error } \Delta S_{\text{UCCSD}} = \frac{|\Delta S_{\text{UCCSDTQ}}|}{|\Delta S_{\text{UCCSD}}|} \times 100. \tag{S15}$$

Table S1: $H_2$ energy and entropy differences relative to CNEO-FCI. Energy differences are given in Hartree and $S \equiv S(\text{e})$. Column labels 2 - 7 are given by Eqs. (S10) – (S15).

| Bond Distance (Å) | $\Delta E_{\text{UCCSDTQ}}$ | $\Delta E_{\text{UCCSD}}$ | % Error $\Delta E_{\text{UCCSD}}$ | $\Delta S_{\text{UCCSDTQ}}$ | $\Delta S_{\text{UCCSD}}$ | % Error $\Delta S_{\text{UCCSD}}$ |
|---|---|---|---|---|---|---|
| 0.40000 | 1.094e-04 | -9.495e-04 | 1.152e+01 | 1.452e-05 | 5.553e-03 | 2.615e-01 |
| 0.49246 | 3.056e-05 | -5.098e-04 | 5.994e+00 | 4.921e-06 | 3.831e-03 | 1.285e-01 |
| 0.60804 | 6.521e-06 | -2.954e-04 | 2.208e+00 | 6.667e-07 | 2.739e-03 | 2.434e-02 |
| 0.76985 | 6.208e-07 | -2.282e-04 | 2.720e-01 | 1.342e-07 | 2.419e-03 | 5.548e-03 |
| 1.00101 | 4.819e-09 | -2.697e-04 | 1.787e-03 | 9.620e-08 | 3.050e-03 | 3.154e-03 |
| 1.25528 | 1.120e-07 | -3.805e-04 | 2.944e-02 | -1.960e-07 | 4.706e-03 | 4.165e-03 |
| 1.53266 | 1.353e-07 | -5.309e-04 | 2.550e-02 | -4.980e-08 | 7.103e-03 | 7.011e-04 |
| 2.01809 | 3.911e-08 | -6.861e-04 | 5.700e-03 | 2.000e-09 | 8.968e-03 | 2.230e-05 |
| 2.52663 | 2.770e-09 | -6.991e-04 | 3.963e-04 | -2.355e-07 | 7.280e-03 | 3.235e-03 |
| 3.49749 | -1.399e-11 | -7.365e-04 | 1.899e-06 | 5.280e-09 | 6.169e-03 | 8.558e-05 |
| 4.19095 | -8.064e-11 | -7.510e-04 | 1.074e-05 | 3.160e-08 | 6.174e-03 | 5.119e-04 |
| 5.00000 | -1.992e-11 | -7.578e-04 | 2.629e-06 | -2.530e-09 | 6.226e-03 | 4.064e-05 |

Table S2: HD energy and entropy differences relative to CNEO-FCI. Energy differences are given in Hartree and $S \equiv S(\mathrm{e})$. Column labels 2 - 7 are given by Eqs. (S10) – (S15).

| Bond Distance (Å) | $\Delta E_{\mathrm{UCCSDTQ}}$ | $\Delta E_{\mathrm{UCCSD}}$ | % Error $\Delta E_{\mathrm{UCCSD}}$ | $\Delta S_{\mathrm{UCCSDTQ}}$ | $\Delta S_{\mathrm{UCCSD}}$ | % Error $\Delta S_{\mathrm{UCCSD}}$ |
|---|---|---|---|---|---|---|
| 0.40000 | 1.103e-04 | -7.743e-04 | 1.424e+01 | 1.327e-05 | 4.798e-03 | 2.765e-01 |
| 0.49246 | 3.040e-05 | -4.125e-04 | 7.369e+00 | 4.151e-06 | 3.251e-03 | 1.277e-01 |
| 0.60804 | 6.372e-06 | -2.397e-04 | 2.658e+00 | 7.177e-07 | 2.306e-03 | 3.112e-02 |
| 0.76985 | 5.760e-07 | -1.863e-04 | 3.092e-01 | 5.280e-08 | 2.031e-03 | 2.600e-03 |
| 1.00101 | 7.930e-09 | -2.214e-04 | 3.582e-03 | 1.700e-09 | 2.572e-03 | 6.609e-05 |
| 1.25528 | 1.175e-07 | -3.134e-04 | 3.748e-02 | -9.110e-08 | 4.001e-03 | 2.277e-03 |
| 1.53266 | 1.360e-07 | -4.366e-04 | 3.115e-02 | 1.327e-07 | 6.054e-03 | 2.192e-03 |
| 2.01809 | 3.809e-08 | -5.551e-04 | 6.861e-03 | 2.236e-07 | 7.548e-03 | 2.962e-03 |
| 2.52663 | 2.768e-09 | -5.564e-04 | 4.975e-04 | 4.515e-07 | 6.014e-03 | 7.508e-03 |
| 3.49749 | -1.455e-11 | -5.819e-04 | 2.501e-06 | 7.618e-08 | 5.051e-03 | 1.508e-03 |
| 4.19095 | -1.706e-11 | -5.929e-04 | 2.877e-06 | -2.500e-09 | 5.053e-03 | 4.948e-05 |
| 5.00000 | -1.566e-11 | -5.982e-04 | 2.618e-06 | 3.256e-08 | 5.095e-03 | 6.390e-04 |

Table S3: $D_2$ energy and entropy differences relative to CNEO-FCI. Energy differences are given in Hartree and $S \equiv S(\mathrm{e})$. Column labels 2 - 7 are given by Eqs. (S10) – (S15).

| Bond Distance (Å) | $\Delta E_{\mathrm{UCCSDTQ}}$ | $\Delta E_{\mathrm{UCCSD}}$ | % Error $\Delta E_{\mathrm{UCCSD}}$ | $\Delta S_{\mathrm{UCCSDTQ}}$ | $\Delta S_{\mathrm{UCCSD}}$ | % Error $\Delta S_{\mathrm{UCCSD}}$ |
|---|---|---|---|---|---|---|
| 0.40000 | 1.105e-04 | -6.267e-04 | 1.764e+01 | 1.164e-05 | 4.121e-03 | 2.824e-01 |
| 0.49246 | 3.009e-05 | -3.298e-04 | 9.123e+00 | 2.849e-06 | 2.743e-03 | 1.039e-01 |
| 0.60804 | 6.192e-06 | -1.897e-04 | 3.265e+00 | 6.120e-07 | 1.914e-03 | 3.197e-02 |
| 0.76985 | 5.285e-07 | -1.451e-04 | 3.641e-01 | 1.867e-07 | 1.657e-03 | 1.127e-02 |
| 1.00101 | 1.172e-08 | -1.713e-04 | 6.838e-03 | -3.700e-08 | 2.087e-03 | 1.773e-03 |
| 1.25528 | 1.233e-07 | -2.434e-04 | 5.067e-02 | -1.833e-07 | 3.269e-03 | 5.607e-03 |
| 1.53266 | 1.366e-07 | -3.387e-04 | 4.034e-02 | 7.910e-08 | 4.963e-03 | 1.594e-03 |
| 2.01809 | 3.705e-08 | -4.213e-04 | 8.793e-03 | -2.760e-08 | 6.092e-03 | 4.530e-04 |
| 2.52663 | 2.639e-09 | -4.118e-04 | 6.408e-04 | -5.157e-08 | 4.735e-03 | 1.089e-03 |
| 3.49749 | -1.679e-10 | -4.262e-04 | 3.940e-05 | -1.200e-08 | 3.925e-03 | 3.057e-04 |
| 4.19095 | -1.211e-11 | -4.339e-04 | 2.791e-06 | 5.160e-09 | 3.924e-03 | 1.315e-04 |
| 5.00000 | -1.689e-11 | -4.377e-04 | 3.860e-06 | 1.133e-08 | 3.957e-03 | 2.863e-04 |

We observe that errors at short bond distances are much larger than those near equilibrium and beyond. The value of % Error $\Delta E_{\mathrm{UCCSD}}$ gives the percentage error of the corresponding point in Figure 2 of the main text. In the extreme case of 0.4 Å, these can range between 11% ($H_2$) to 17% ($D_2$). As the bond length is stretched to more reasonable distances, the errors fall considerably, and at 0.6 Å the error is already between 2% ($H_2$) to 3% ($D_2$). Close to equilibrium it falls to $< 1\%$ for all species, and remains very low for the remainder of the sampled points. We note that these percentage error values are for an energy *difference*, and that the percent error in the value of the CNEO-UCCSDTQ energy itself due to the retraint procedure is always $<< 1\%$ even in the most extreme cases.

Finally, we mention that these values can be significantly improved by adjusting the value of $\mu$. The chosen value of $\mu$ used in this work gives accurate results near equilibrium while also allowing the calculations to reliably converge in the extreme cases of compressed bond lengths. For a more tailored study involving chemical phenomena near equilibrium only, it is therefore very likely that a larger value of $\mu$ could be employed (and convergence reliably achieved) to attain even more accurate results.
